# Construction of a preoperative prehabilitation program for elderly esophageal cancer patients

**DOI:** 10.3389/fonc.2025.1694667

**Published:** 2025-12-10

**Authors:** Xuejiao Chen, Kun Zhou, Fengjuan Cai, Zihao Liu, Caifeng Luo, Xiao Shao

**Affiliations:** 1 Oncology Department, The Affiliated Suqian Hospital of Xuzhou Medical University, Suqian, Jiangsu, China; 2 Interventional and Geriatric Department, The Affiliated Suqian Hospital of Xuzhou Medical University, Suqian, Jiangsu, China; 3 Endoscopy Center, Jiangsu Province (Suqian) Hospital, Suqian, Jiangsu, China; 4 School of Medicine, Jiangsu University, Zhenjiang, Jiangsu, China

**Keywords:** elderly, esophageal cancer, prehabilitation, Delphi method, program construction

## Abstract

**Objective:**

To develop a prehabilitation program for elderly patients with esophageal cancer before surgery.

**Methods:**

Relevant studies on prehabilitation for esophageal cancer were comprehensively searched and analyzed to summarize the best available evidence. A preliminary prehabilitation program was drafted through group discussion and subsequently refined via two rounds of expert consultation using the Delphi method, resulting in the final program.

**Results:**

The prehabilitation program for elderly patients with esophageal cancer before surgery includes 5 first-level items, 12 second-level items, and 35 third-level items.

**Conclusion:**

The prehabilitation program for elderly patients with esophageal cancer before surgery demonstrates certain scientific validity and reliability, providing a reference for healthcare professionals in guiding patients’ prehabilitation.

## 1 Introduction

The Global Cancer Epidemiology Report (2024) indicates that in 2022, both the incidence and mortality rates of esophageal cancer in China ranked among the highest worldwide (1), with elderly patients accounting for over 70% of cases (2), imposing substantial medical and economic burdens on society and families. Surgery, as the primary treatment modality for esophageal cancer (3), involves a “window period” between tumor diagnosis and operative intervention. During this period, elderly patients commonly experience cancer-related issues such as decreased physical activity, increased nutritional risk, and prominent psychological problems (4–6). In addition, age-related decline in physiological functions leads to reduced surgical tolerance, higher perioperative mortality risk, and increased incidence of complications (7, 8). Moreover, preoperative immunosuppression, slower metabolic rate, and frequent multimorbidity (9, 10) result in a poor preoperative status, which adversely affects postoperative recovery. Therefore, effectively improving the preoperative functional status of elderly patients with esophageal cancer to enhance prognosis and quality of life warrants urgent attention.

In response to the poor preoperative functional status commonly observed in elderly patients with esophageal cancer, “prehabilitation” has been proposed as a scientific and effective approach. Prehabilitation, based on Enhanced Recovery After Surgery (ERAS), is a comprehensive preoperative intervention that includes exercise, nutritional assessment, and supplementation, as well as anxiety management (11). It has been widely applied in colorectal surgery, cardiac surgery, and other disciplines both domestically and internationally (12, 13). However, no scientific and standardised preoperative prehabilitation program has yet been developed. Therefore, this study, conducted a literature search and screening, summarized best evidence, and expert consultation, aims to establish a preoperative prehabilitation program for elderly patients with esophageal cancer, thereby providing a foundation for the standardized implementation of such programs.

## 2 Materials and methods

The materials and methods were performed as previously described in our study (14). The following is the detailed content:

### 2.1 Problem identification

The evidence-based question of this study was determined using the PIPOST framework (15). P (Population): patients undergoing esophageal cancer surgery; I (Intervention): prehabilitation program measures aimed at improving the physiological and psychological status and quality of life of surgical patients with esophageal cancer; P (Professional): clinicians, nurses, nutritionists, psychological counsellors, rehabilitation physicians, and anesthesiologists; O (Outcome): nutritional indicators, six-minute walk distance, anxiety and depression status, quality of life, postoperative length of hospital stay, and complications; S (Setting): thoracic surgery department; Tx(Type of evidence): primarily systematic reviews, guidelines, expert consensus, randomized controlled trials (RCTs), and quasi-experimental studies.

### 2.2 Literature search strategy

**Figure 1:**
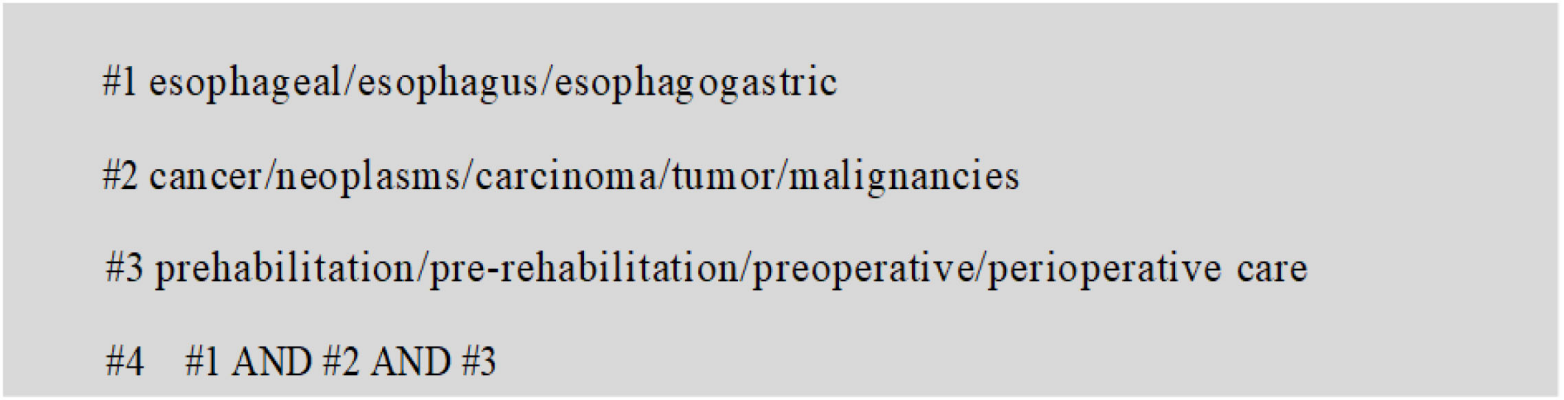
Literature search strategy.

Drawing on the “5S” evidence model, a top-down search was conducted in both domestic and international databases, including UpToDate, BMJ Best Practice, National Institute for Health and Care Excellence (NICE), The Cochrane Library, Web of Science, PubMed, CINAHL, National Guideline Clearinghouse (NGC), Registered Nurses’ Association of Ontario (RNAO), China National Knowledge Infrastructure (CNKI), and Wanfang Data. Literature related to prehabilitation in patients undergoing esophageal cancer surgery was retrieved, with the search period set from database inception to 31 May 2022. Search terms included: “esophageal/esophagus/esophagogastric”, “cancer/neoplasms/carcinoma/tumor/malignancies”, and “prehabilitation/pre-rehabilitation/preoperative/perioperative care”. Taking PubMed as an example, its specific retrieval strategy is shown in **Figure 1**.

**Figure 2:**
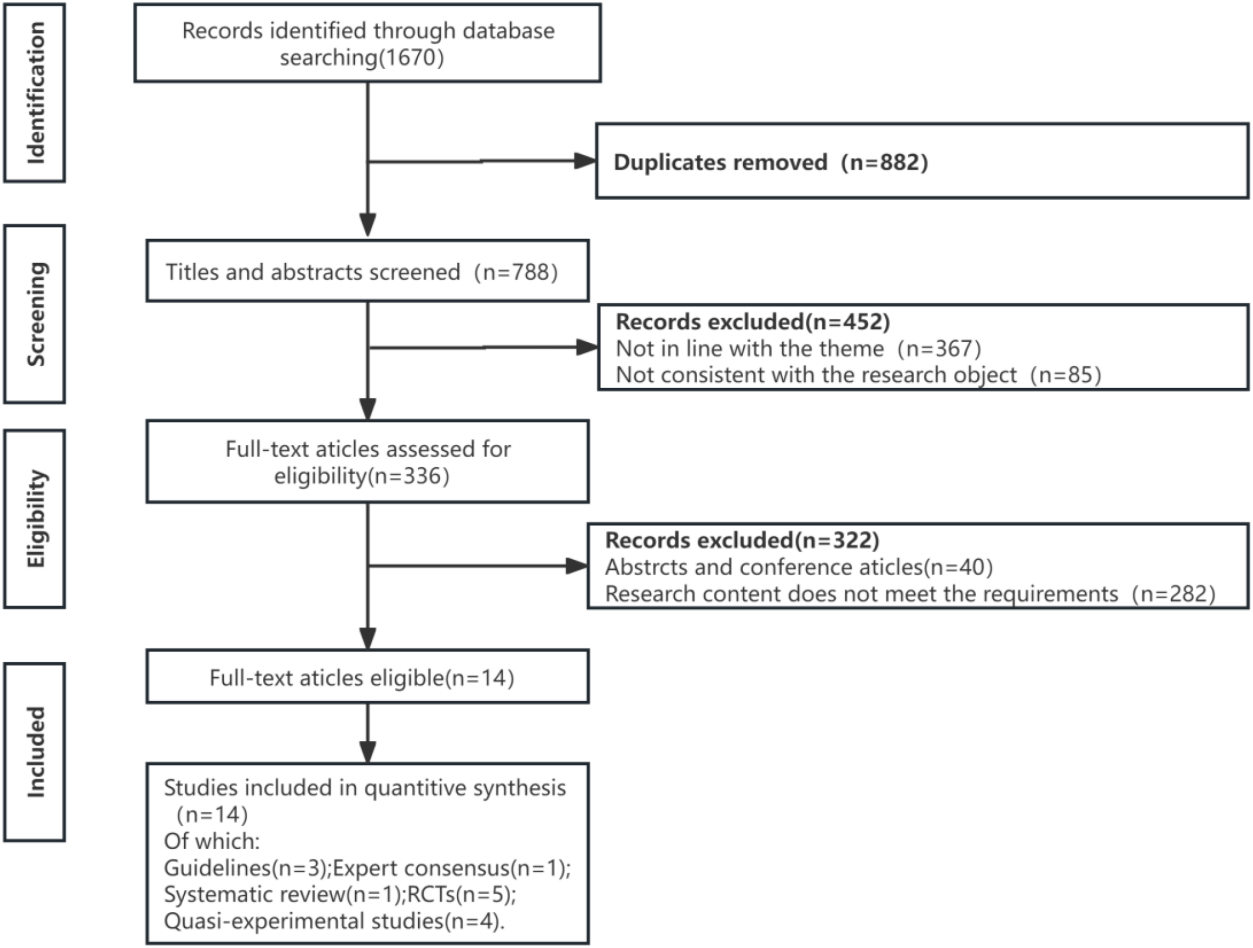
The literature selection flowchart.

Literature inclusion criteria: (1) Study population: elderly patients undergoing esophageal cancer surgery; (2) Studies related to prehabilitation; (3) Outcome indicators including six-minute walk distance, nutritional indicators, anxiety and depression status, quality of life, postoperative length of hospital stay, and complications; (4) All the search period was set from the inception of each database to May 31, 2022. We selected research published within the past decade (i.e., from approximately May 2012 to May 2022). (5) Publications in both Chinese and English. Exclusion criteria: (1) Studies with pediatric or adolescent patients as the research population; (2) Studies with inaccessible full text, or those lacking research design details or abstracts; (3) Studies with insufficient methodological quality. The literature selection flowchart is shown in **Figure 2**.

### 2.3 Quality appraisal and evidence extraction

The quality of the included literature was appraised according to the following standards: (1) Guidelines: evaluated using the Appraisal of Guidelines for Research and Evaluation II (AGREE II) instrument (16); (2) Expert consensus: assessed according to the quality appraisal criteria of the Joanna Briggs Institute (JBI) Centre for Evidence-Based Healthcare, Australia (2016) (17); (3) Systematic reviews: appraised using the AMSTAR tool (18); (4) Randomized controlled trials and quasi-experimental studies: evaluated with the JBI quality appraisal criteria (2016, Australia) (19, 20). Two postgraduate students trained in evidence-based nursing independently conducted the quality appraisal. In case of disagreement, consensus was reached through discussion with an expert in evidence-based nursing until a unified judgement was achieved. When evidence from different sources was inconsistent, preference was given to the most recent publications with higher methodological quality. Evidence grading and extraction were conducted according to the JBI levels of evidence and the grades of recommendation system (2014, Australia) (21).

### 2.4 Drafting of the preoperative prehabilitation program and expert consultation

Based on the integrated evidence and in consideration of the characteristics of elderly patients with esophageal cancer, the research team invited six experts with extensive clinical and nursing experience in thoracic surgery, rehabilitation, anesthesiology, nutrition, psychology, and nursing management. Two group meetings were held, each lasting 1–2 hours, to discuss the preliminary draft of the preoperative prehabilitation regimen for elderly esophageal cancer patients that had been developed through a literature review. During the meetings, participants independently expressed their opinions and engaged in collective discussion. Recommendations supported by strong evidence were directly incorporated, whereas components with weak or insufficient evidence were initially formulated based on expert consensus. Members of the research team documented the meeting proceedings, and after the expert consultations, they collated and analyzed the expert feedback and revised the initial draft of the regimen accordingly.

### 2.5 Statistical methods

SPSS 26.0 and Excel were used for data entry, summarization, and analysis. Measurement data were expressed as mean ± standard deviation ( $\bar{x}\pm s$), while categorical data were described using frequency and percentage. Expert enthusiasm was evaluated by the proportion of experts who provided constructive opinions and the questionnaire response rate. The authority coefficient of experts (Cr) was determined by calculating the arithmetic mean of the judgement coefficient (Ca) and the familiarity coefficient (Cs). A Cr value greater than 0.7 is generally considered indicative of higher reliability of research findings (22). The degree of consensus among experts was assessed using the coefficient of variation (CV) and Kendall’s coefficient of concordance (Kendall’s W) (23).

## 3 Results

### 3.1 Literature screening and quality appraisal

A total of 3 guidelines (13, 24, 25), 1 expert consensus (8), 1 systematic review (26), 5 randomised controlled trials (RCTs) (27–31), and 4 quasi-experimental studies (32–35) were finally included in this study. Among the guidelines, 2 were rated grade A and 1 grade B. The single expert consensus was appraised as “Yes” for all items. In the systematic review, all items were rated as “Yes” except for item 4, “Were publication characteristics, such as grey literature, considered in the inclusion criteria?”, which was rated as “No”. For the 5 RCTs, quality appraisal results are shown in **Table 1**. For the 4 quasi-experimental studies, all appraisal items were rated as “Yes”.

**Table 1:** Quality appraisal of randomised controlled trials.

Evaluation criteria	Minnella 2018 (30)	Valkenet 2018 (29)	Allen 2022 (27)	Yamana 2015 (31)	Guo Zhong 2018 (28)
1. Was true randomization achieved?	Yes	Yes	Yes	Yes	Yes
2. Was allocation concealment performed?	Yes	Yes	Yes	Yes	Unclear
3. Were baseline characteristics comparable between groups?	Yes	Yes	Yes	Yes	Yes
4. Was blinding of participants implemented?	No	No	No	No	Unclear
5. Was blinding of intervention providers implemented?	No	No	No	No	Unclear
6. Was blinding of outcome assessors implemented?	Yes	Yes	Yes	Unclear	Unclear
7. Apart from the intervention under study, were other measures identical across groups?	Yes	Yes	Yes	Yes	Yes
8. Was follow-up complete, and were strategies applied to address loss to follow-up?	Yes	Yes	Yes	Yes	Yes
9. Were all randomized participants included in the analysis?	No	Yes	Yes	Yes	Yes
10. Were outcome measures assessed using consistent methods across groups?	Yes	Yes	Yes	Yes	Yes
11. Were the outcome assessment methods reliable?	Yes	Yes	Yes	Yes	Yes
12. Were appropriate methods used for data analysis?	Yes	Yes	Yes	Yes	Yes
13. Was the study scientifically designed, and did the implementation deviate from a standard RCT?	Yes	Yes	Yes	Yes	Yes

### 3.2 General information of experts

Before the consultation, we established the inclusion criteria for experts: ① From a tertiary comprehensive hospital; ② Having a bachelor’s degree or above; ③ Engaged in relevant professional work for more than 10 years; ④ Having intermediate or higher professional titles; ⑤ Voluntarily participating in this inquiry. After discussing the actual situation, a total of 16 experts participated in two rounds of consultation in this study. These experts were staff members from 9 tertiary grade A hospitals and 3 tertiary hospitals in Jiangsu Province, with research and clinical expertise covering thoracic surgery, rehabilitation, anaesthesiology, nutrition, psychology, and nursing. General information is presented in **Table 2**.

**Table 2:** General information of consultation experts (n = 16).

Item	Category	Frequency (n)	Percentage (%)
Gender	Male	7	43.75
	Female	9	56.25
Age	30-39	3	18.75
	40-49	7	43.75
	≥50	6	37.50
Education	Bachelor’s	10	62.50
	Master’s	6	37.50
Work field	Clinical nursing	7	43.75
	Clinical medicine	4	25.00
	Clinical nutrition	2	12.50
	Clinical psychology	1	6.25
	Anesthesiology	1	6.25
	Nursing management	1	6.25

### 3.3 Expert enthusiasm, authority, and consensus

In the two rounds of consultation, 16 questionnaires were distributed and all were returned. The judgment coefficient (Ca) of the experts was 0.918 in both rounds, and the familiarity coefficient (Cs) was 0.875, resulting in an authority coefficient (Cr) of 0.897 for both rounds. The degree of consensus among experts was assessed using the coefficient of variation (CV) and Kendall’s coefficient of concordance (Kendall’s W). The CVs for importance ratings of each item in the two rounds ranged from 0 to 0.19 and 0 to 0.18, respectively, while the CVs for feasibility ratings ranged from 0 to 0.17. Kendall’s W ranged from 0.213 to 0.384. These results indicate that the experts demonstrated high enthusiasm, good authority, and satisfactory consensus.

### 3.4 Consultation results

After two rounds of consultation, the experts reached a relatively unified consensus, and a preoperative prehabilitation program for elderly patients with esophageal cancer was finalised. As shown in **Table 3**, the program comprises 5 first-level items, 12 second-level items, and 35 third-level items.

**Table 3:** Preoperative prehabilitation program for elderly patients with esophageal cancer.

First-level item	Second-level item	Third-level item	Importance score (mean ± SD)	Coefficient of variation (CV)
1 Organizational management	1-1 Establishment of prehabilitation team	1-1-1 Establish a prehabilitation team composed of multidisciplinary members, including thoracic surgeons, anesthesiologists, nurses, psychological counsellors, nutritionists, and postgraduate students.	4.88 ± 0.34	0.07
		1-1-2 Clarify the qualifications, roles, and responsibilities of team members: ① Thoracic surgeons are responsible for disease diagnosis and treatment; ② Rehabilitation specialists and anesthesiologists formulate personalized exercise rehabilitation plans; ③ Nutritionists assess patients’ nutritional status, analyze requirements for nutrients and energy, and develop tailored nutritional prescriptions; ④ Psychological counsellors provide mental support and consultation for those in need; ⑤ Nursing masters and nurses deliver health education, including guidance on psychology, exercise, and diet. The team provides guidance on exercise and diet, evaluates whether patients’ conditions improve as a result of the interventions, and collects, organizes, and analyses the relevant data.	4.81 ± 0.40	0.08
		1-1-3 Training and assessment: regularly organise training and assessment for prehabilitation team members. In addition to evaluating skills and knowledge, the assessment also examines evidence-based thinking.	4.75 ± 0.45	0.09
		1-1-4 Quality control: the team supervises and implements the prehabilitation program, defines the workflow for each stage, develops improvement plans for the program, and continuously enhances the overall quality.	4.44 ± 0.51	0.11
2 Screening and assessment	2-1 General condition and disease assessment	2-1-1 Age, sex, American Society of Anesthesiologists (ASA) classification, body mass index (BMI), and use of walking aids;	4.81 ± 0.40	0.08
		2-1-2 Disease-related symptoms (e.g., dysphagia, appetite, nausea, vomiting), signs, severity, treatment status, and type of surgery;	4.44 ± 0.51	0.11
		2-1-3 Surgical history and medication history, and their potential impact on prehabilitation implementation;	4.31 ± 0.79	0.18
		2-1-4 Imaging examinations: chest and abdominal ultrasound, CT, MRI, etc.	4.75 ± 0.45	0.09
		2-1-5 Laboratory tests: tumor markers including cytokeratin fragment 19 (CYFRA21-1), carcinoembryonic antigen (CEA), squamous cell carcinoma antigen (SCC), tissue polypeptide-specific antigen (TPS), complete blood count, biochemical profile, etc.	4.75 ± 0.45	0.09
	2-2 Cardiopulmonary function and frailty assessment	2-2-1 Cardiopulmonary fitness: six-minute walk distance (6MWD)	3.94 ± 0.44	0.11
		2-2-2 Pulmonary function: forced vital capacity (FVC) and forced expiratory volume in 1 second (FEV_1_)	4.81 ± 0.40	0.08
		2-2-3 Frailty assessment using the Fried Phenotype (FP) scale	4.06 ± 0.25	0.06
	2-3. Nutrition screening and assessment	2-3-1 Nutritional risk screening at initial diagnosis using the Nutritional Risk Screening 2002 (NRS 2002)	4.69 ± 0.48	0.10
		2-3-2 Patients at nutritional risk undergo immediate nutritional assessment using the Patient-Generated Subjective Global Assessment (PG-SGA)	4.88 ± 0.34	0.07
		2-3-3 Patients with malnutrition receive comprehensive nutritional assessment within 24 hours, including dietary survey and laboratory indicators (e.g., hemoglobin, albumin, globulin, lymphocytes, transferrin, prealbumin)	4.81 ± 0.40	0.08
	2-4. Psychological, sleep, and cognitive screening	2-4-1 Psychological status: assessed using the Hospital Anxiety and Depression Scale (HADS)	4.56 ± 0.51	0.11
		2-4-2 Sleep status: assessed using the General Sleep Disturbance Scale (GSDS)	4.88 ± 0.34	0.07
		2-4-3 Cognitive status: assessed using the Mini-Cog	4.38 ± 0.50	0.11
	2-5. Social support assessment	2-5-1 Patients’ and family members’ education level, per capita monthly household income, and social support	4.75 ± 0.45	0.09
3. Planning	3-1. Formulation of prehabilitation intervention program	3-1-1 Establish short-term and long-term goals for preoperative prehabilitation interventions, and dynamically adjust the intervention plan based on patient response.	3.94 ± 0.44	0.11
		3-1-2 Exercise training: ① Low-risk patients: a. Inspiratory muscle training (IMT): patient seated, inhale rapidly and exhale slowly, starting at resistance level 0 and gradually increasing; 30 repetitions per set, 1–2 sets/day. b. Aerobic exercise: brisk walking, cycling, jogging, etc., using a modified Borg scale of 13–16; 30–40 min per session (including 5 min warm-up, 20–30 min target intensity, 5 min cooldown), 3 sessions per week. c. Resistance training: seated or supine, using resistance bands or body weight (e.g., seated knee raises, chest expansion against resistance); intensity based on 8–15 repetition maximum (RM), 2 sets of 10 repetitions per session, 2 sessions per week. ② Medium- and high-risk patients: after comprehensive evaluation, if the universal program above is applied, it should be conducted under supervision of a physiotherapist, ensuring cardiopulmonary function is monitored; personalised programs can be applied if necessary.	4.06 ± 0.25	0.06
		3-1-3 Nutritional optimization: ① Patients with malnutrition receive interventions according to comprehensive nutritional assessment and the “five-step ladder” principle while controlling symptoms: a. Diet plus nutrition education (baseline); b. Diet plus oral nutritional supplements; c. Total enteral nutrition if oral intake insufficient; d. Partial enteral plus partial parenteral nutrition for complete inability to eat; e. Total parenteral nutrition for patients with gastrointestinal obstruction, etc. ② High-quality protein supplementation: 1.2–1.5 g/kg per day; whey protein intake 1 hour after daily exercise. ③ Determine intake method, quantity, and frequency. ④ Decide the route of nutrition supplementation (prefer oral first, then enteral, then parenteral), start time, and duration. Total enteral nutrition via a nasogastric tube if oral intake is insufficient and no obstruction exists; if obstruction is present, consider percutaneous endoscopic gastrostomy/jejunostomy (PEG/PEJ) for nutritional access”. ⑤ Preoperative day infusion: 500 mL of 5% glucose normal saline intravenously to improve metabolism and reduce insulin resistance. ⑥ The target for nutritional intervention is to achieve a weight gain of 0.5-1 kg per week, or to attenuate weight loss to less than 2% of body weight per week. The ultimate goal is to restore or maintain BMI within the normal range (18.5-24.9 kg/m²) prior to surgery.	4.44 ± 0.51	0.11
		3-1-4 Psychological support: ① Encourage patients to express feelings; listen patiently and provide reassurance; use supportive gestures such as holding hands or gentle shoulder taps. ② Encourage self-regulation techniques, e.g., listening to music before sleep, performing home relaxation exercises, or meditation. ③ Psychological counsellors provide interventions for patients with depression or anxiety risk as indicated by HADS.	4.88 ± 0.34	0.07
		④ For severe sleep disturbances, analyze risk factors. if depression or anxiety is present, manage accordingly, including medication if prescribed. ⑤ Cognitive training for patients with cognitive impairment.		
		3-1-5 Other interventions: ① Smoking cessation: encourage early cessation and provide pharmacologic support per medical advice. ② Correct anemia: iron supplementation for iron-deficiency anemia; vitamin B12 or folic acid for megaloblastic anemia. ③ Pain management: assess pain level thoroughly and administer analgesics according to medical guidance.	4.06 ± 0.25	0.06
4Prehabilitation Intervention	4-1 Patient and Caregiver Education	4-1-1 A written informed consent is signed with the patient before the implementation of the prehabilitation intervention	3.94 ± 0.44	0.11
		4-1-2 Patients and family members are instructed and trained in the specific contents of the prehabilitation program, and their cooperation is obtained: ① Training formats are diversified, such as multimedia materials, health education manuals, individual counselling, and peer support. ② Training contents include the rationale for prehabilitation, specific objectives and principles, and the significance and purpose of implementing the prehabilitation program. ③ Training tools involve not only traditional approaches (e.g., brochures, charts), but also prehabilitation videos to provide more intuitive content and facilitate comprehension.	4.63 ± 0.50	0.11
	4-2 program Implementation	4-2-1 The prehabilitation intervention is implemented according to the formulated program, with dynamic adjustments made based on the patient’s condition	4.81 ± 0.40	0.08
	4-3 Record Improvement	4-3-1 The implementation of the prehabilitation program is regularly documented, the patient’s compliance is evaluated, and timely guidance and correction are provided	4.88 ± 0.34	0.07
		4-3-2 The effects of the prehabilitation intervention, adverse reactions, and complications are recorded	4.19 ± 0.40	0.10
55 Monitoring, Evaluation and Feedback	5-1 Follow-up and Monitoring	5-1-1 An electronic record is established, which mainly includes basic information such as patients’ economic status, medical insurance type, age, and sex, as well as patients’ psychological and nutritional status, prehabilitation plan, and disease stage.	4.50 ± 0.52	0.12
		5-1-2 Members of the prehabilitation team are responsible for monitoring and follow-up.	3.94 ± 0.44	0.11
		5-1-3 Follow-up methods include home visits, telephone follow-up, and real-time monitoring via remote online platforms. The specific method is mainly determined according to patients’ basic conditions.	4.69 ± 0.48	0.10
		5-1-4 Follow-up time and content: Nurses evaluate the implementation of prehabilitation by reviewing daily prehabilitation logs, photos, and videos uploaded via WeChat. Monitoring also includes whether complications occur: ① Exercise: excessive cardiopulmonary load, soft tissue or muscle injury. Pre-exercise evaluation is required, and progressive training is performed according to individualized protocols within patients’ tolerance. ② Nutrition: refeeding syndrome, electrolyte and metabolic disorders, infections, and gastrointestinal symptoms. Regular monitoring of patients’ nutritional status and laboratory indicators is performed to prevent complications. ③ Psychology: continuous assessment of severe anxiety, depression, or sleep disorders. Psychological consultants provide timely interventions. Patients’ tolerance and adherence, psychological experience, and nursing needs of both patients and caregivers are also investigated.	4.88 ± 0.34	0.07
	5-2 Evaluation and Feedback	5-2-1 Outcome evaluation: At key time points including the decision for surgery, one day before surgery, 7 days after surgery, and 4 weeks after surgery, patients are evaluated for 6MWD, laboratory indicators, anxiety and depression (HADS), and health-related quality of life (SF-36) to assess the effect of prehabilitation.	4.06 ± 0.25	0.06
		5-2-2 Dynamic feedback: Continuation of the original program: if the prehabilitation demonstrates satisfactory outcomes, the original program is continued. Termination of the program: the program is terminated in cases of severe complications, hospital transfer, or withdrawal from surgery	4.44 ± 0.51	0.11

## 4 Discussion

### 4.1 Reliability and scientific validity of the preoperative prehabilitation program for elderly patients with esophageal cancer

This study was designed on the basis of a preliminary survey of the current situation and guided by evidence-based principles. A comprehensive literature search related to the topic was conducted in accordance with the “5S model”, and the relevant evidence was systematically summarized to initially formulate a preoperative prehabilitation program for elderly patients with esophageal cancer, demonstrating a certain degree of scientific validity. As the retrieved evidence involved cultural differences between domestic and international contexts, a process of localization was required before clinical application. Therefore, 16 experts in the relevant field were invited for consultation. These experts had more than 10 years of working experience, all held at least a bachelor’s degree, and were employed in tertiary general hospitals with abundant clinical experience and solid theoretical knowledge. After two rounds of consultation, the analysis showed a positive coefficient and authority coefficient of 100% and 0.897, respectively. The coefficients of variation for each item ranged from 0 to 0.19, while the overall Kendall’s W ranged from 0.213 to 0.384 (*P*< 0.05). The coordination and consistency of expert opinions on each item were relatively high. These findings indicated that the preoperative prehabilitation program developed in this study possessed high reliability.

### 4.2 The prehabilitation program for elderly patients with esophageal cancer demonstrates strong specificity

Compared with existing programs, the preoperative prehabilitation program constructed in this study presents the following advantages: (1) Intervention population: A randomized controlled trial (RCT) conducted by Minnella et al. (30) on patients with esophagogastric cancer demonstrated the safety and feasibility of comprehensive prehabilitation. However, elderly patients with esophageal cancer often have a high incidence of frailty and poor nutritional status (36, 37), which reduces their tolerance to surgery. Therefore, once esophageal cancer is diagnosed, a preoperative prehabilitation program should be initiated immediately in patients who meet the surgical indications. The present program targets elderly patients with oesophageal cancer, providing individualized prehabilitation interventions before surgery and implementing the optimal scheme as much as possible to improve postoperative outcomes. (2) Intervention timing: The study by Lambert et al. (38) indicated that although the implementation period of prehabilitation varied among patients with different types of cancer, linear regression analysis showed no significant association between the duration of prehabilitation and outcome indicators. Nevertheless, elderly patients with esophageal cancer usually present poor postoperative mobility and nutritional status, and thus the “window period” before surgery should be fully utilized for prehabilitation training. In addition, since both patients and their attending physicians generally expect early surgery after diagnosis, and in accordance with the Expert Consensus on Prehabilitation Management for Thoracic Surgery Based on Enhanced Recovery After Surgery (8), the duration of prehabilitation in this study was defined as from pathological confirmation to the day before surgery (at least one week), this duration is slightly shorter than the 8-20 weeks of Machado’s meta-analysis (39) on prehabilitation for colon cancer and lung cancer (3). Intervention content: The prehabilitation program developed in this study was based on multidisciplinary collaboration, allowing individualized plans to be designed and applied for patients. A detailed plan combining nutritional supplementation and moderate exercise has been developed for elderly patients with esophageal cancer to improve their preoperative nutritional status and functional abilities. During implementation, healthcare providers performed regular supervision and follow-up to ensure safety. Furthermore, the program was refined with detailed planning regarding the timing, frequency, and intensity of prehabilitation, as well as the methods of follow-up assessment, thereby demonstrating strong feasibility and scientific validity.

## 5 Summary

Based on the synthesis of best available evidence, this study employed the Delphi expert consultation method to develop a prehabilitation program for elderly patients undergoing surgery for esophageal cancer. The program comprises five domains: organizational management, screening and assessment, planning, prehabilitation interventions, and monitoring with feedback. The program demonstrates a certain degree of scientific validity and reliability. It provides clinical healthcare providers with a reference for implementing individualized prehabilitation management in elderly patients with esophageal cancer. However, compared with younger patients, elderly individuals have relatively diminished physiological and psychological functions. Therefore, for safety considerations, comprehensive monitoring of all patient indicators is recommended throughout the implementation of the prehabilitation program.

## Data Availability

The raw data supporting the conclusions of this article will be made available by the authors, without undue reservation.
